# Melamine Impairs Renal and Vascular Function in Rats

**DOI:** 10.1038/srep28041

**Published:** 2016-06-21

**Authors:** Xiao Yu Tian, Wing Tak Wong, Chi Wai Lau, Yi-Xiang Wang, Wai San Cheang, Jian Liu, Ye Lu, Huihui Huang, Yin Xia, Zhen Yu Chen, Chuen-Shing Mok, Chau-Ming Lau, Yu Huang

**Affiliations:** 1 School of Biomedical Sciences, Institute of Vascular Medicine and Li Ka Shing Institute of Health Sciences, Chinese University of Hong Kong, Hong Kong; 2 Department of Diagnostic Radiology and Organ Imaging, Chinese University of Hong Kong, Hong Kong; 3 School of Life Sciences, Chinese University of Hong Kong, Hong Kong; 4 Hong Kong Government Laboratory, Hong Kong

## Abstract

Melamine incident, linked to nephrotoxicity and kidney stone in infants previously exposed to melamine-contaminated milk products, was unprecedentedly grave in China in 2008 as little was known about the mechanistic process leading to renal dysfunction in affected children. This study investigates whether neonatal ingestion of melamine leads to renal and vascular dysfunction in adulthood; and whether ingestion of melamine in pregnant rats leads to renal dysfunction in their offspring. A combination of approaches employed includes functional studies in rat renal arteries, renal blood flow measurement by functional magnetic resonance imaging, assay for pro-inflammatory and fibrotic biomarkers, immunohistochemistry, and detection of plasma and renal melamine. We provide mechanistic evidence showing for the first time that melamine reduces renal blood flow and impairs renal and vascular function associated with overexpression of inflammatory markers, transforming growth factor-β1, bone morphogenic protein 4 and cyclooxygenase-2 in kidney and renal vasculature. Melamine also induces renal inflammation and fibrosis. More importantly, melamine causes nephropathies in offsprings from pregnant rat exposed to melamine during pregnancy, as well as in neonatal rat exposed to melamine afterbirth, thus supporting the clinical observations of kidney stone and acute renal failure in infants consuming melamine-contaminated milk products.

Contamination of milk products with melamine in China in 2008 caused a widespread public outcry and health scare as excessive exposure to melamine leads to the formation of bilateral kidney stones causing severe obstruction of the urinary tract, which results in hospitalization of thousands. Acute renal failure associated with melamine has cost lives of infants although the renal toxicity of melamine in humans has not been reported before. Melamine (1,3,5-triazine-2,4,6-triamine), used industrially to manufacture fire-retardant plastics, adhesive and fertilizers, is derived from urea. Melamine contains a high level of nitrogen, which is deliberately added to milk products to boost their protein contents on the standardized test that determines nitrogen content as a surrogate for protein. Following the pet food incident in 2007, the United States Food and Drug Administration (FDA) released an Interim Melamine and Analogues Safety/Risk Assessment and recommended a safe daily intake of 0.63 mg·kg^−1^ body weight·day^−1^ for melamine, while the European Food Safety Authority has provisionally recommended 0.5 mg·kg^−1^ body weight·day^−1^ for the total of melamine and its analogues as an acceptable level of daily intake. The FDA issued an official statement that there is “very low risks to human health from consuming meat from hogs and chickens known to have been fed animal feed supplemented with pet food scraps that contained melamine and melamine-related compounds”. There have been very limited data concerning the nephrotoxic effects of melamine in humans until the outbreak of massive illnesses in infants consuming melamine-contaminated dairy products.

Animal studies show that crystal deposition is in the renal distal tubules and collecting ducts which eventually forming calculi. Melamine is not metabolized in the body and it is rapidly excreted in urine with a half-life in plasma for approximately 3 hours. Melamine and its analogue, cyanuric acid alone are of a low acute toxicity; however, the outbreak of acute renal failure in cats and dogs associated with the consumption of contaminated pet food suggests that co-ingestion of melamine and cyanuric acid could cause more severe renal toxicity^1^. Melamine and cyanuric acid may form a complex called melamine cyanurate, which has a very low solubility due to its highly ordered lattice structure held together by multiple hydrogen bonds between molecules^2^,^3^. This complex is thought to be involved in the formation of kidney crystals^4^,^5^. Since most of the events resulted from the ingestion of food contaminated with melamine and cyanuric acid, melamine and cyanuric acid might be absorbed through the gastrointestinal tract, distributed systemically and accumulated especially in the distal renal tubules, and finally lead to progressive tubular blockage and renal damage^6^,^7^. However, it is basically unknown where the melamine cyanurate complex is primarily formed in kidney in relation to their accumulated concentrations and how the pH value of the renal tubular fluid may impact the process involved in kidney stone formation associated with low solubility of melamine cyanurate. In the present study, we investigate whether neonatal ingestion of melamine leads to renal and vascular dysfunction in adulthood; and whether ingestion of melamine in pregnant rats leads to renal dysfunction in their offspring.

## Results

### Basic parameters in melamine-treated rats

**Figure 1:**
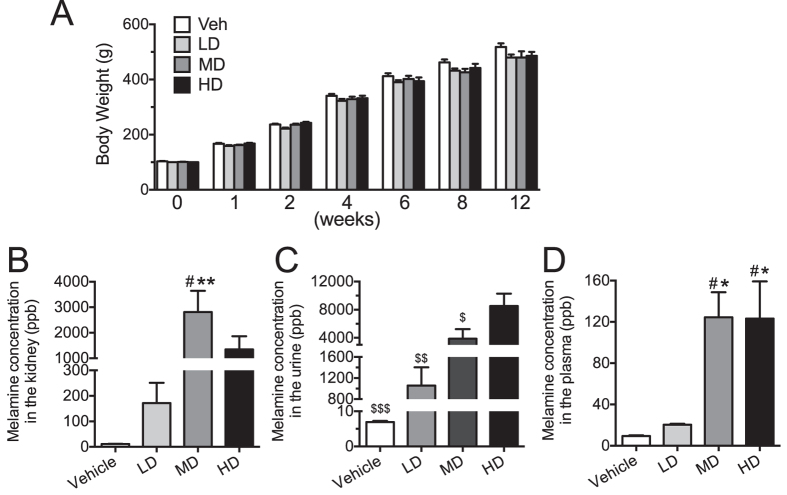
Effects of melamine consumption on body weights and melamine accumulation in plasma and kidney samples. (**A**) Melamine consumption did not significantly affect the body weights of the rats in both the low dosage (LD), medium dosage (MD) and high dosage (HD) group compared to the vehicle-treated group (Veh). (**B,C**) Melamine treatment dose-dependently increased melamine content in plasma and kidney tissues. Results are means ± SEM of 6–8 rats. *p < 0.05 vs vehicle control. ^#^p < 0.05 vs LD. ^$^p < 0.05; ^$$^p < 0.01; ^$$$^p < 0.001 vs HD.

Three-month oral consumption of melamine in drinking water at three dosages (60, 300 and 600 mg·kg^−1^ body weight·day^−1^) in SD adult male rats did not change the body weights (Fig. 1A). Melamine consumption (at medium and high dose) significantly elevated the concentration of melamine in both plasma (Fig. 1B) and kidney samples (Fig. 1C). Melamine ingestion did not affect the systolic blood pressures, heart weight and weight of kidney on both sides did not change significantly (data not shown).

### Impaired endothelial function in renal arteries of melamine-treated rats in a dose-dependent manner

**Figure 2:**
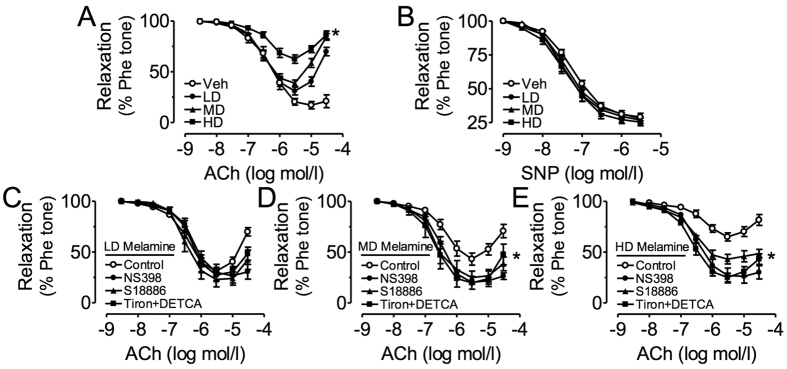
Effects of melamine consumption on acetycholine (ACh)-induced endothelium-dependent relaxations (EDRs). (**A**) Melamine consumption dose-dependently reduced the ACh-induced EDRs in intralobal renal arteries without affecting the SNP-induced endothelium-independent relaxations (**B**). (**C–E**) NS398, S18886 and Tiron plus DETCA improved the impaired ACh-induced EDRs in renal arteries of the melamine-treated rats. Results are means ± SEM of 6–8 rats. *p < 0.05 vs vehicle control. LD: low dose; MD: medium dose; HD: high dose.

In isolated SD rat renal arteries constricted with phenylephrine, the cumulative addition of acetylcholine (ACh) caused endothelium-dependent relaxations (Fig. 2A). Chronic consumption of melamine impaired the ACh-induced relaxations with reduction in maximum relaxation and higher concentrations of ACh turned relaxation into contractile responses (Fig. 2A). By contrast, the endothelium-independent relaxations in response to sodium nitroprusside (SNP) were identical in renal arteries isolated from vehicle control and melamine-treated rats (Fig. 2B). To dissect the possible mechanisms responsible for the impaired relaxations, the roles of COX-2, thromboxane prostanoid (TP) receptor and reactive oxygen species (ROS) were examined. In renal arteries from rats treated with melamine at three different doses, NS398 (COX-2 inhibitor), S18886 (TP receptor antagonist) and tiron plus DETCA (ROS inhibitor and scavenger) markedly improved ACh-induced relaxations in medium and high dose treatment groups (Fig. 2D,E) and reduced the contractile phase in all the three-dosage groups (Fig. 2C–E).

**Figure 3:**
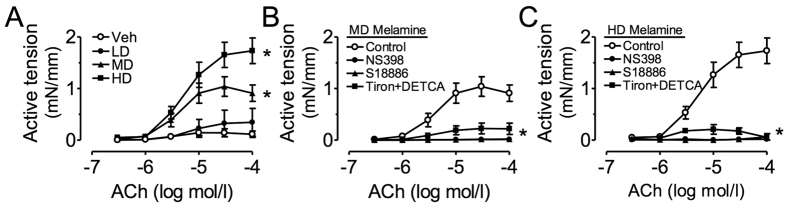
Effects of melamine consumption on acetycholine (ACh)-induced endothelium-dependent contractions (EDCs). (**A**) Melamine consumption dose-dependently increased the ACh-induced EDCs in intralobal renal arteries in the presence of L-NAME. (**B,C**) NS398, S18886 and Tiron plus DETCA inhibited the ACh-induced EDCs in renal arteries of the melamine-treated rats. Results are means ± SEM of 6–8 rats. *p < 0.05 vs vehicle control. LD: low dose; MD: medium dose; HD: high dose.

We also tested the impact of melamine consumption on the appearance of endothelium-dependent contractions (EDCs) in response to ACh in renal arteries following acute treatment with NOS inhibitor, L-NAME. In non-treated vehicle control rats, ACh-induced contractions were negligible and low-dose melamine ingestion caused a small contraction (Fig. 3A). On the other hand, medium and high dose of melamine triggered large EDCs (Fig. 3A). Treatment with NS398, S18886, or tiron plus DETCA abolished the EDCs in renal arteries from medium- and high-dose melamine-treated rats (Fig. 3B,C).

### Impaired renal cortical blood flow in melamine-treated rats

**Figure 4:**
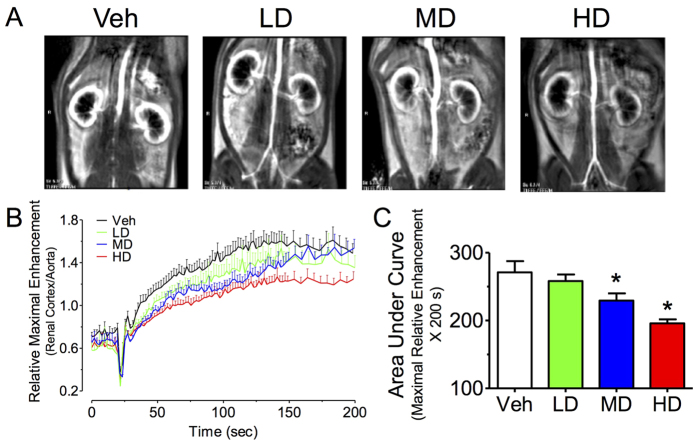
Effects of melamine consumption on renal blood flow. (**A**) Representative images showing the renal cortices and aortae representing maximal relative enhancement. (**B,C**) The cortical enhancement was reduced in melamine-treated rats in a dose-dependent manner. Results are means ± SEM of 4–6 rats. *p < 0.05 vs vehicle control. LD: low dose; MD: medium dose; HD: high dose.

Originally we have tested the efficacy of using computerized tomography (CT) to visualize the renal stone formation upon melamine administration. However, we found no detectable sizes of renal stone and since the images captured were not clear due to the poor resolution of the CT machine, we had switched to use functional magnetic resonance imaging (fMRI) to detect the changes in morphology of kidneys and measure the renal blood flow. Regions of interest (ROI) were drawn in the renal cortex of both kidneys. Fig. 4A shows the representative images of the renal cortices and aortae showing maximal relative enhancement. The cortical enhancement was reduced in melamine-treated rats in a dose-dependent manner (Fig. 4B,C).

### Increased pro-inflammatory and pro-fibrotic markers in renal arteries and kidney of melamine-treated rats

**Figure 5:**
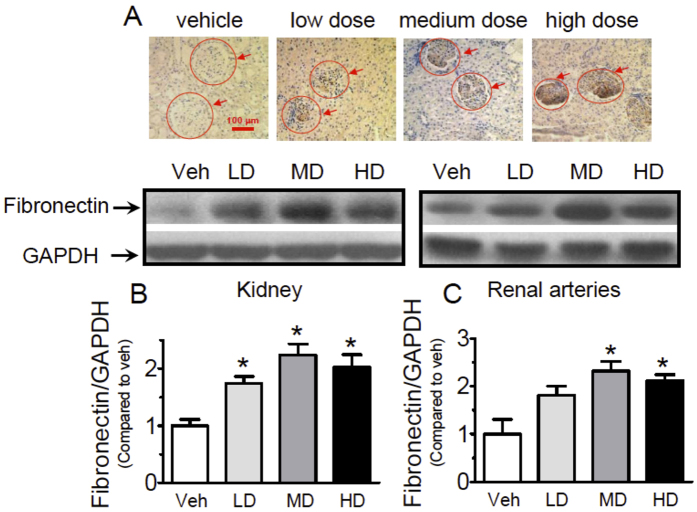
Effects of melamine consumption on fibronectin expressions in renal arteries and kidneys. (**A**) Representative images of immunohistochemistry staining of kidney showing melamine treatment led to markedly increased expression of fibronectin in glomeruli in a dose-dependent manner. (**B,C**) Western blotting showing elevated fibronectin levels in both kidneys and renal arteries. Results are means ± SEM of 4 rats. *p < 0.05 vs vehicle control. LD: low dose; MD: medium dose; HD: high dose.

**Figure 6:**
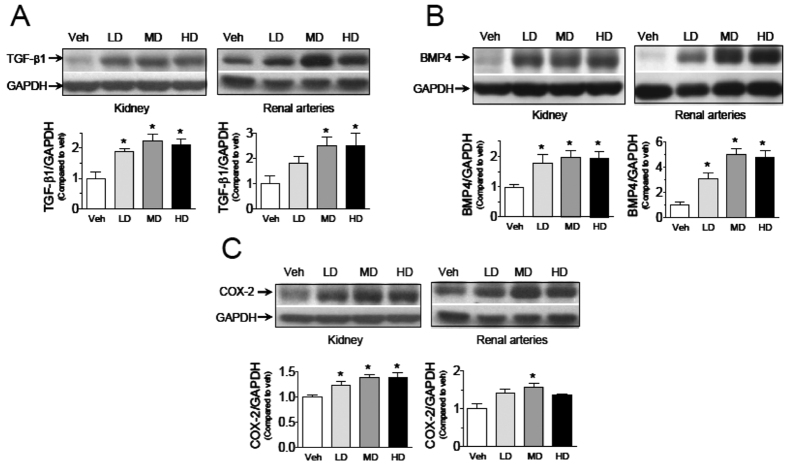
Effects of melamine consumption on pro-inflammatory markers in renal arteries and kidneys. Increased expression of (**A**) TGF-β1, (**B**) BMP4, and (**C**) COX-2 in kidney and renal arteries in melamine-treated rats. Results are means ± SEM of 4 rats. *p < 0.05 vs vehicle control. LD: low dose; MD: medium dose; HD: high dose.

Immunohistochemistry staining of kidney showed that melamine treatment led to markedly increased expression of fibronectin in glomeruli in a dose-dependent manner (Fig. 5A), suggesting the remodeling of kidney and arteries were taking place. This effect was further confirmed by Western blotting showing elevated fibronectin levels in both kidneys (Fig. 5B) and renal arteries (Fig. 5C). The expression of TGF-β1 in kidney and renal arteries in melamine-treated rats were also upregulated (Fig. 6A), further indicating the remodeling response to melamine treatment. Furthermore, increased expression of bone morphogenic protein 4 (BMP4) (Fig. 6B) and cyclooxygenase-2 (COX-2) (Fig. 6C) in both kidney and renal arteries following chronic melamine consumption suggesting both tissues may undergo an inflammatory process.

### Consumption of melamine in pregnant rats led to renovascular dysfunction in their offspring

**Figure 7:**
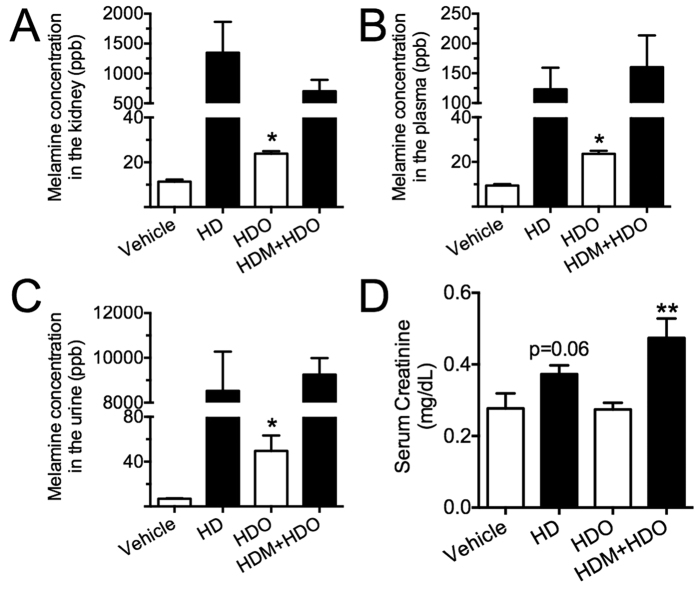
Consumption of melamine in pregnant rats increased melamine concentration in their offsprings. (**A–C**) Melamine content was increased in kidney, plasma and urine from the male offspring (continuing to receive high-dose melamine for three months) from high-dose melamine-treated mothers (HDM + HDO), and the male offspring (stopped receiving melamine) from high-dose melamine-treated mothers (HDO), compared to high dose melamine (HD) and vehicle control. (**D**) Serum creatinine level from all 4 groups of rats. *p < 0.05, p = 0.06 vs Vehicle. **p < 0.01 vs HDO.

**Figure 8:**
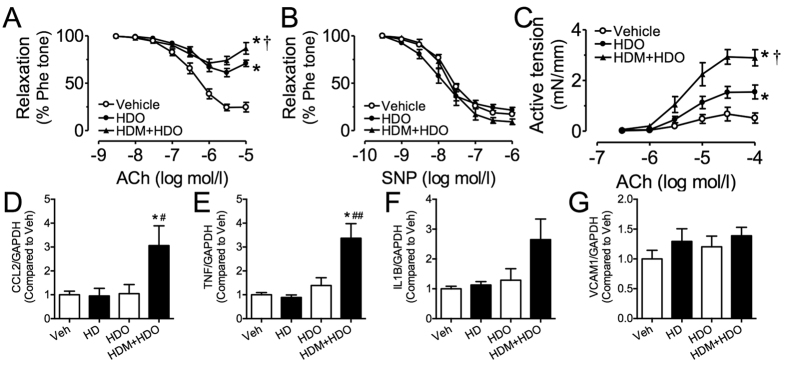
Melamine exposure before birth potentiated renovascular dysfunction and inflammation. (**A**) The ACh-induced endothelium-dependent relaxations (EDRs) were greatly impaired in renal arteries from HDO group as compared with vehicle control. The ACh-induced EDRs were further aggravated from HDM + HDO group. (**B**) SNP-induced endothelium-independent relaxations were comparable in the three groups. (**C**) The ACh-induced EDCs were greater in renal arteries from HDO group as compared with vehicle control. And the EDCs further increased from HDM + HDO group. Results are means ± SEM of 4–6 rats. *p < 0.05 vs vehicle control; ^†^p < 0.05 vs HDO. (**D–G**) mRNA expressions of CCL2, TNF, IL1β, and VCAM1 in kidneys. *p < 0.05 vs HD; ^#^p < 0.05, ^##^p < 0.01 vs HDO.

The male offspring given rise by high-dose melamine-treated mothers kept on receiving high-dose melamine at 600 mg·kg^−1^ body weight·day^−1^ for another three months (HDM + HDO). HDM + HDO rats had markedly increased concentration of melamine in both plasma (Fig. 7B) and kidney samples (Fig. 7A) as compared with the offspring from the same high-dose melamine-treated mothers but stopped receiving melamine (HDO). Compared to rats receiving high dose melamine (HD), HDM + HDO rats have similar melamine concentrations in kidney, plasma, and urine (Fig. 7A–C), but slightly higher serum creatinine (Fig. 7D). Interestingly, although HDO rats did not receive melamine treatment, due to their exposure to melamine before birth, the melamine contents in kidney, plasma, and urine remained significantly higher than rats from vehicle group even after 3 months. The ACh-induced EDRs were greatly impaired in renal arteries from HDO rats as compared with vehicle control (Fig. 8A). The ACh-induced EDRs were further aggravated in renal arteries from HDM + HDO rats than those from HD rats (Fig. 8A). SNP-induced endothelium-independent relaxations were comparable in the three groups (Fig. 8B). The ACh-induced EDCs were enhanced in renal arteries from HDO rats as compared with vehicle control. And the EDCs further increased in HDM + HDO rats (Fig. 8C), indicating that embryonic exposure to melamine may have long term harmful effect on renovascular function. Accordingly, several pro-inflammatory markers including CCL2, TNF, and IL1β, although not increased in kidneys from HD rats, were all upregulated in HDM + HDO rats (Fig. 8D–F), showing that chronic inflammation in the kidney due to melamine ingestion was potentiated by embryonic exposure to melamine.

## Discussion

The major findings of the present study include long term exposure of melamine impaired renal vascular function in intralobal renal arteries and renal blood flow. There is clear sign of renal fibrosis in melamine-treated rats corresponding to an increase in accumulation of melamine in blood and kidneys of rats treated with melamine. The present study thus provides some useful information for clinicians and researchers to have more understanding on the pathophysiological events related to the melamine incident.

In the present study, we found that long-term exposure to melamine (>3 months) showed impaired renal blood flow and renal vascular function suggesting a potential harmful effect of melamine after long-term exposure. Indeed, recent studies also showed a positive correlation of the concentration of urinary melamine and the risk of kidney stone^8^, although the health impact of melamine in adults have not been fully studied. In order to investigate the effect of melamine consumption, the dose-dependent effects of melamine on renal blood flow, vascular function, inflammatory responses and fibrosis were studied. Melamine dose-dependently reduced renal blood flow and vasodilatory function of renal arteries. Vascular function of intralobal renal arteries is important for maintaining renal blood perfusion and regulates glomerular filtration rates thus affecting renal function. Previous studies showed that melamine causes renal toxicity as manifested by presence of melamine stones, histopathological changes showing nephropathy, and impaired renal function indicated by increased serum creatinine level. Our study is the first to use renal blood flow test to show the effect of melamine on renal function, and the results suggested that renovascular function may be a target of melamine accumulation in the kidney apart from renal tubules.

In order to further study the underlying mechanisms of melamine-impaired renal function, we showed that the impact of melamine might be related to pro-inflammatory pathways mediated by cyclooxygenase activation and renal fibrosis. Data from vasoreactivity measurement and Western blotting showed the involvement of cyclooxygenase-thromboxane receptor which enhanced vasoconstriction while reduced vasodilatation in intralobal renal arteries which contributes to reduced renal blood perfusion. COX-2-derived PGF_2α_ which acts on TP receptor is the major endothelium-dependent contracting factor produced by endothelial cells^9^,^10^. This COX-2-PGF_2α_-TP receptor is important in inducing endothelial dysfunction in renovascular hypertensive rats and spontaneous hypertensive rats^11^, and in BMP4-treated mice^9^. In addition, the upregulation and activation of COX-2 requires ROS. ROS inhibitor improves endothelium-dependent vasodilatation and inhibits endothelium-dependent vasoconstriction. Increased ROS production is invariably important in all forms of chronic kidney diseases, mainly driven by the local renin-angiotensin system and oxidant-producing enzymes (NOX), and also in acute nephrotoxicity induced by various chemicals^12^,^13^,^14^. COX-2 is expressed in renal arteries, podocytes, and the loop of Henle. COX-2 and PGE_2_ regulate water reabsorption, so increased COX-2 and PGE_2_ production impairs renal capacity to concentrate urine, while promotes inflammation in obstructive nephropathy. Increased COX-2 expression is also found in other kidney diseases such as acute kidney injury, diabetic nephropathy, renal artery stenosis, etc., all indicating that COX-2 is an important enzyme in both homeostasis and dysfunction of kidney^15^.

In addition, we also demonstrate upregulation of fibronectin, TGF-β1, and BMP4 in the kidney, suggesting that melamine intake may trigger the development of renal fibrosis. TGF-β1, BMP4, and the downstream Smad pathway are not only involved in kidney disease, but also in vascular inflammation, remodeling, and fibrosis^16^,^17^. These findings provided potential therapeutic targets for managing melamine-related kidney diseases.

Of importance, we found that exposure of mothers to melamine exaggerated melamine-induced renovascular dysfunction, and inflammation in offspring, indicating a maternal transfer of melamine through gestation. Both transfer routes through placenta or milk were validated by other studies^18^,^19^. Importantly, we showed that melamine is able to retain in the offspring after 3 months, indicating a potential long-term renal damage or likely a predisposition to other kidney diseases. However, we do not have the data to show whether and how embryo is exposed to melamine and also to cyanuric acid, although higher dose is considered to be both embryotoxic and maternotoxic^20^,^21^,^22^. These findings raise the health concern of monitoring melamine-contaminated food for mothers.

To conclude, the present study provides mechanistic evidence on the harmful effects of consumption of melamine on renal function. We have defined the dose-damage relationship of melamine, roles of inflammation or increased oxidative stress and subsequently decreased nitric oxide bioavailability in vascular and renal dysfunction and their relation to the development of interstitial fibrosis and eventual renal damage. The present study also provides evidence revealing how melamine causes nephropathies in neonatal animals and impact on postnatal animals resulting from exposure to melamine during pregnancy.

## Materials and Methods

All animal experiments were approved by the Animal Experimental Ethics Committee of the Chinese University of Hong Kong, in accordance with the approved guidelines for animal care and long-term experimental protocol, under the license issued by the Department of Health of Hong Kong SAR. General lab safety and chemical and biological experimental safety approvals were obtained for this project from the University Safety Office of the Chinese University of Hong Kong. All methods were carried out in accordance with the relevant guidelines from the Chinese University of Hong Kong or the Department of Health HKSAR.

### Animals

All animal experiments were approved by the Animal Experimental Ethics Committee of the Chinese University of Hong Kong, in accordance with the approved guidelines for animal care and long-term experimental protocol, under the license issued by the Department of Health of Hong Kong SAR. Sprague Dawley (SD) rats were used in the present study. Rats were housed in a temperature-controlled holding room (22–23 °C) with a 12-h light/dark cycle, and fed a standard chow and water. Urine was collected using a metabolic cage. In order to study the dose-dependent effects of melamine, male 4-week-old rats were given melamine dissolved in water freely for 3 months at three dosages (60, 300 and 600 mg·kg^−1^ body weight·day^−1^) while in the control group the rats were given normal access to water. To study the effects of melamine on offspring from melamine-treated pregnant rats, 2-month old female rats were given melamine at a high dose (600 mg·kg^−1^ body weight·day^−1^) for 2 weeks before they were mated with male rats and continued during gestation. The offspring given rise by these melamine-treated mothers were further divided into melamine-treated group (HDM + HDO) that the male pups kept on receiving high-dose melamine at 600 mg·kg^−1^ body weight·day^−1^ for another three months and vehicle-treated group (HDO) that the pups stopped receiving melamine. The volume of water consumption was monitored daily and body weight was measured weekly to adjust the amount of melamine dissolved in drinking water in order to obtain the approximate melamine intake at three dosages (60, 300 and 600 mg·kg^−1^ body weight·day^−1^). After sacrifice of rats, weights of the heart and the kidneys were measured. Systolic blood pressure was measured by the tail-cuff method and blood pressure was calculated from the average of 5 successive recordings. Serum creatinine was measured by creatinine assay kit (Abcam) according to manufacturer’s protocol.

### Renal artery preparation and vascular reactivity assay

Rats were sacrificed by CO_2_ suffocation and intralobal renal arteries were removed and placed in ice-cold Krebs solution (mmol/l): 119 NaCl, 4.7 KCl, 2.5 CaCl_2_, 1 MgCl_2_, 25 NaHCO_3_, 1.2 KH_2_PO_4_, and 11 D-glucose. Arteries were prepared and changes of isometric tension were recorded^23^. The organ chamber was filled with 5 ml Krebs solution and gassed by 95% O_2_-5% CO_2_ at 37 °C (pH ~7.4). Each renal artery segment was stretched to 2.5 mN, an optimal tension, and then allowed to stabilize for 90 min before the start of each experiment. Each ring was initially contracted by 60 mmol/l K^+^ -containing Krebs solution. Endothelium-dependent relaxations (EDRs) to acetylcholine (ACh, 0.003 to 10 μmol/l) and endothelium-independent relaxations to sodium nitroprusside (SNP, 0.001 to 10 μmol/l) were examined in arteries pre-contracted with phenylephrine (1 μmol/l). Endothelium-dependent contractions (EDCs) to acetylcholine (ACh, 0.3 to 100 μmol/l) were examined in the presence of 100 μmol/l N^G^-nitro-L-arginine methyl ester (L-NAME). EDRs and EDCs to ACh in renal arteries from melamine-treated rats were studied in control and in the presence of each of the following inhibitors (30-min incubation): cyclooxyenase-2 (COX-2) inhibitor NS398 (3 μmol/l), thromboxane prostanoid (TP) receptor antagonist S18886 (0.1 μmol/l) or ROS inhibitors tiron (1 mmol/l) plus DETCA (100 μmol/l).

### Magnetic resonance imaging (MRI) analysis

All MRI studies were performed using a 3 T clinical whole-body imaging system (Achieva, Philips Healthcare, Best, The Netherlands). Methods were modified according to previous studies^24^,^25^,^26^. After anesthesia, animals were positioned supine and placed in a coil for data acquisition. The MR image acquisition of the rat urinary system included high resolution T2 weighted axial plane anatomical examination; high resolution T1 weighted coronal plane anatomical examination; and dynamic contrast enhanced examination in coronal plane. Axial anatomical examinations were acquired with the following parameters: multiple slice turbo spine echo sequence, TR/TE/flip angle = 2359 ms/120 ms/90°, field of view = 60 mm × 81 mm × 30 mm, the acquisition voxel size was 0.41 mm × 0.41 mm × 1.50 mm, and the reconstructed voxel size was 0.17 mm × 0.17 mm × 1.5 mm. Coronal anatomical examinations were acquired with the following parameters: 3D gradient echo sequence with fat suppression, TR/TE/flip angle = 4.4 ms/2.2 ms/10°, field of view = 80 mm × 80 mm × 18 mm, the acquisition voxel size was 0.50 mm × 0.50 mm × 1.00 mm and the reconstructed voxel size was 0.28 mm × 0.28 mm × 0.50 mm. The contrast-enhanced examinations were acquired with the following parameters: 3D gradient echo sequence, TR/TE/flip angle = 6.8 ms/2.3 ms/35°, field of view = 80 mm × 80 mm × 12 mm, the acquisition voxel size was 0.61 mm × 0.75 mm × 3.00 mm and the reconstructed voxel size was 0.31 mm × 0.31 mm × 1.5 mm. MRI contrast agent was Gd-DOTA (Guerbet Group, Roissy CDG cedex, France). A dose of 0.075 mmol/kg was intravenously injected through tail vein as a rapid bolus in less than 1 s after initial baseline 10 acquisitions as quick bolus and followed by a flush of 0.5 ml normal saline. Dynamic scan was stopped when the contrast agent was excreted and clearly visible in the bilateral ureters. The reconstructed MR images were transferred to a radiological workstation (Extended Workspace, Philips, Best, Netherlands) for off-line analysis. Anatomical images were read by a radiologist with animal research experiences. For analysis of dynamic data, regions of interest (ROIs) were manually drawn over left and right kidneys. The ROIs of the renal cortex were drawn in all rats. These ROIs were used on the perfusion-weighted data to generate time signal intensity curves.

### Histology and immunohistochemistry

SD rats were anesthetized with ketamine hydrochloride (150 mg/kg body weight i.p.) and xylazine (7.5 mg/kg body weight i.p.), then perfused transcardially with 100 ml 0.9% NaCl followed by 250 ml 4% paraformaldehyde in phosphate buffer saline (pH 7.4). Both kidneys were dissected out and fixed in fixative overnight at 4 °C, then dehydrated, processed and embedded in paraffin. Five-micrometer sections were stained with periodic acid-Schiff or napthol AS-D chloroacetate esterase. For immunostaining, primary antibody against fibronectin was used to target its expression in the glomeruli.

### Western blotting

Renal arteries or kidney samples were homogenized with ice-cold RIPA lysis buffer containing 1 μg/ml leupeptin, 5 μg/ml aprotonin, 100 μg/ml PMSF, 1 mmol/l sodium orthovanadate, 1 mmol/l EDTA, 1 mmol/l EGTA, 1 mmol/l sodium fluoride and 2 μg/ml β-glycerolphosphate. The protein concentration was determined by Lowry method (Bio-rad) and aliquots of 50 μg of the total proteins were separated on 10% SDS-poly-acrylamide gel. Proteins were then transferred to immobilon-P polyvinylidene difluoride (PVDF) membrane (Millipore). Membranes were blocked with 5% non-fat milk in TBS-T and subsequently exposed to primary antibodies including transforming growth factor (TGF)-β1 (Abcam), fibronectin (Abcam), bone morphogenic protein 4 (Sigma-Aldrich), and cyclooxygenase-2 (Cayman chemicals) followed by HRP-conjugated secondary antibody and developed by chemiluminescence.

### Quantitative PCR

Rats were perfused with PBS-heparin, and whole kidney was snap frozen and grinded into power in liquid nitrogen. 10% of the renal tissue was further homogenized in Trizol to extract total RNA. cDNA synthesis was done using High-Capacity cDNA Reverse Transcription Kit (ABI) and mRNA expression of each gene was by quantitative PCR using SYBR green master mix and ViiA7 Real Time PCR system. Primers include: ratCCL2-Fwd: 5′-CTT CTG GGC CTG TTG TTC AC-3′ and 5′-GCC AGT GAA TGA GTA GCA GC-3′; ratIL1B-Fwd: 5′-TCT GAC CCA TGT GAG CTG AA-3′ and Rev: 5′-TGT CGT TGC TTG TCT CTC CT-3′; ratVCAM1-Fwd: 5′-TCT GGG AAA CTG GAA AGA GGA-3′ and Rev: 5′-TGG GTA AAC ATC AGG AGC CA-3′; ratTNF-Fwd: 5′-CCA CCA CGC TCT TCT GTC TA-3′ and Rev: 5′-ATC TGA GTG TGA GGG TCT GG-3′; rat GAPDH-Fwd: 5′-GAC ATG CCG CCT GGA GAA AC-3′ and Rev: 5′-AGC CCA GGA TGC CCT TTA GT-3′.

### Analysis of melamine

Melamine was quantified by high performance liquid chromatography (HPLC) method. In brief, a gram of kidney samples was homogenized with 25 ml of acetonitrile:water:diethylamine (50:40:10, v/v) and centrifuged. An aliquot (2.5 ml) of the supernatant was transferred into a glass screw-cap test tube followed by addition of 5.5 ml of acetonitrile. The sample was vortexed and the tube was then centrifuged again. An aliquot (4 ml) of supernatant was then filtered through a 0.8 μmol/l Acrodisc syringe filter into a separate tube and evaporated dry under nitrogen. The dried extract was reconstituted in 490 μl of 80:20 water:acetonitrile. Ten microliters of 10 μg/ml ^15^N-melamine in acetonitrile:water (1:1) was added to the extract, resulting in a sample concentration of 0.10 g/ml. The mixture was then vortexed for 10 s, sonicated for 2 min, and filtered through a 0.45 μm HPLC filter into a 2 ml autosampler vial. A HPLC was used for all analyses. The analytical column is a 150 mm × 4.6 mm i.d., 4 μm Synergi Polar-RP. The mobile phase consists of: (A) 10 mmol/l ammonium acetate in water, (B) acetonitrile, and (C) 0.1% formic acid in water at a flow rate of 0.5 ml/min. Gradient elution was employed: initial mobile phase at 80% A, 15% B, 5% C held for 1 min, and changed to 30% A, 65% B, 5% C at 5 min followed by changing the mobile phase to 5% A, 90% B, 5% C and hold until 7 min. Quantification of all melamine was performed using the internal standard method. Standard solutions were prepared in 80:20 water:acetonitrile at levels of 50, 100, 200, 500, and 1000 ng/ml and used to generate calibration curves. ^15^N-Melamine was included in each standard at 200 ng/ml.

### Chemicals

Chemical used are as follow: ACh, DETCA, L-NAME, melamine, NS398 phenylephrine, tiron, S18886, and SNP (Sigma-Aldrich, St Louis, MO, USA). DETCA and NS397 were dissolved in dimethyl sulfoxide (DMSO) while other drugs were prepared in distilled water. DMSO at 0.1% (v/v) did not affect the vascular reactivity.

### Data Analysis

Results were mean ± SEM from different rats. Concentration-response curves were analyzed by non-linear regression curve fitting using two-way ANOVA followed by Bonferroni *post-hoc* tests (GraphPad Prism software, Version 4.0) to approximate E_max_ as the maximal response and p*D*_2_ as the negative logarithm of the drug concentration that produced 50% of E_max_. The protein expression was quantitated by densitometer and normalized to GAPDH. Images of immunohistochemistry staining were analyzed by ImageJ (NIH). Statistical significance were determined by two-tailed Student’s *t*-test or one-way ANOVA followed by Bonferroni post-hoc tests when more than two treatments were compared. A *P* value of less than 0.05 was regarded as statistically significant.

## Additional Information

**How to cite this article**: Tian, X. Y. *et al.* Melamine Impairs Renal and Vascular Function in Rats. *Sci. Rep.* **6**, 28041; doi: 10.1038/srep28041 (2016).
